# Correction: Zeng et al. High-Performance Silicon Nanowire Array Biosensor for Combined Detection of Colorectal Cancer Biomarkers. *Micromachines* 2025, *16*, 1089

**DOI:** 10.3390/mi16121381

**Published:** 2025-12-05

**Authors:** Jiaye Zeng, Mingbin Liu, Xin Chen, Jintao Yi, Wenhe Liu, Xinjian Qu, Chaoran Liu, Serestina Viriri, Guangguang Yang, Xun Yang, Weichao Yang

**Affiliations:** 1 School of Electronic and Information Engineering, China West Normal University, Nanchong 637002, China; 2 Ministry of Education Engineering Research Center of Smart Microsensors and Microsystems, College of Electronics and Information, Hangzhou Dianzi University, Hangzhou 310018, China; 3 School of Mathematics, Statistics & Computer Science, University of KwaZulu-Natal, Durban 4041, South Africa; 4 School of Electronic and Information Engineering, Foshan University, Foshan 528000, China; guangguangyanguop@gmail.com

Following publication, we noted that the sequence of the corresponding authors and their email addresses was incorrectly listed in the original publication, and a correction is required. With this correction, the Editorial Office and the authors have made the following amendments to the published article [1]:

This correction changes “Weichao Yang ^1,^* and Xun Yang ^1,^*” to “Xun Yang ^1,^* and Weichao Yang ^1,^*”, and “Correspondence: yangwc@cwnu.edu.cn (W.Y.); yangxun@cwnu.edu.cn (X.Y.)” to “Correspondence: yangxun@cwnu.edu.cn (X.Y.); yangwc@cwnu.edu.cn (W.Y.)”.

The authors state that the scientific conclusions are unaffected. This correction was approved by the Academic Editor. The original publication has also been updated.
